# Arthroscopic Shoulder Biceps Suspensionplasty for Hemiplegic Shoulder Painful Inferior Subluxation

**DOI:** 10.1016/j.eats.2023.02.037

**Published:** 2023-05-29

**Authors:** Olivier Bozon, Bertrand Coulet

**Affiliations:** a From the Department of Orthopaedic Surgery, Upper Limb Surgery Unit, Hospital Lapeyronie, CHU Montpellier; b Institut de Neuro-Orthopédie Montpellier INOM, Hospital Lapeyronie, Montpellier, France

## Abstract

Inferior glenohumeral subluxation represents one of the causes of disabling pain in patients with hemiplegia after a stroke. In the case of failure of medical treatment by orthosis or electrical stimulation, surgical treatment of suspensionplasty has been described with good results. We describe here an arthroscopic technique of glenohumeral suspensionplasty by tenodesis of the biceps, proposed in the context of a painful glenohumeral subluxation in patients with hemiplegia.

Hemiplegic shoulder pain is a frequent complication after stroke. It leads to difficulties in rehabilitation, alterations in quality of life, and increased rates of depression.^1^ Various causes of this pain have been identified, such as the presence of associated sensory or motor deficits, spasticity of the shoulder muscles, a lesion of the supraspinatus tendon, tendinitis of the long portion of the biceps, or an inferior glenohumeral subluxation (GHS).^2^ GHS is defined as an abnormal gap between the inferior aspect of the acromion and the superior aspect of the humerus (Fig 1). During the recovery phase after a stroke, loss of muscle tone, particularly of the supraspinatus and deltoid, leads to inferior subluxation of the humeral head and tension on the richly innervated inferior capsule and glenohumeral ligaments. Numerous medical treatments have been described for the management of painful GHS, but no single method has been shown to be superior or to maintain its effectiveness over time.^3^

**Fig 1:**
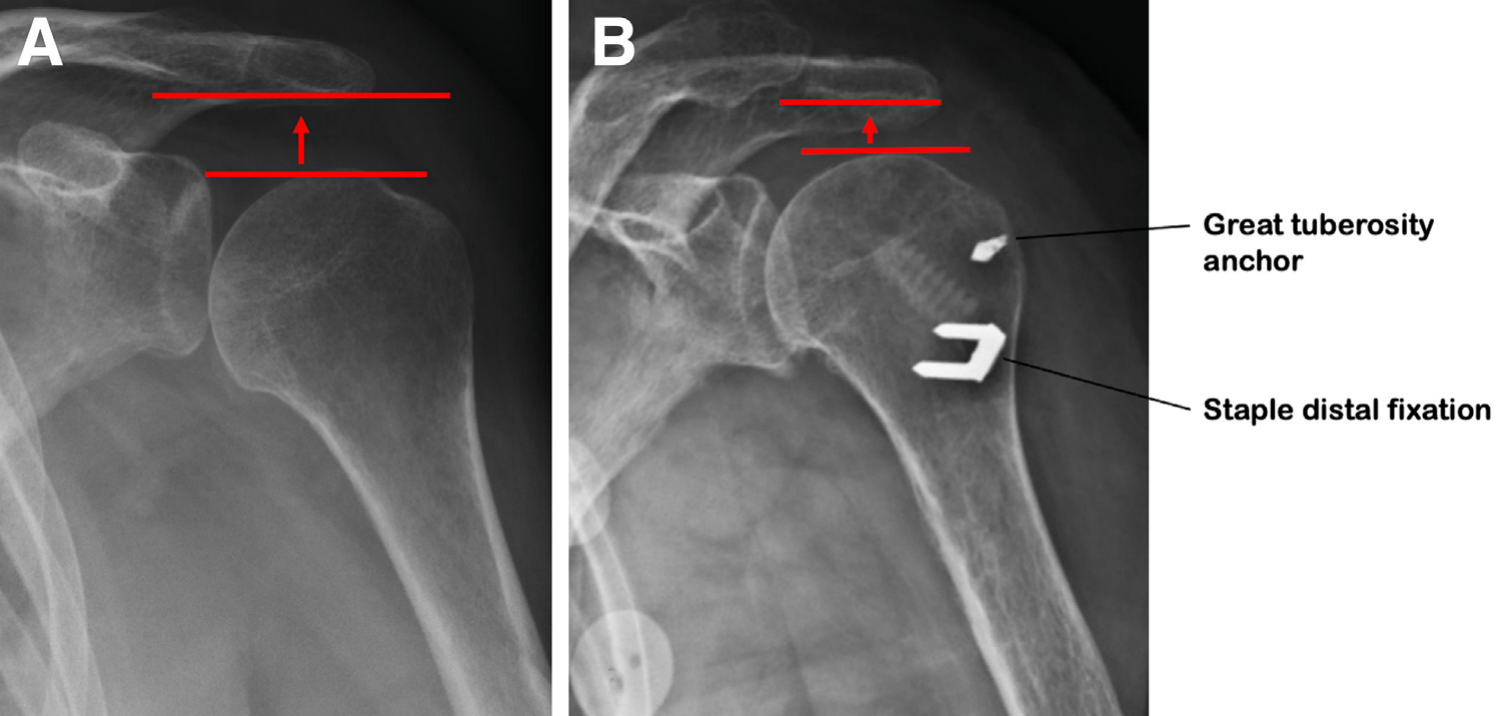
Anteroposterior radiograph of a left shoulder showing inferior subluxation with abnormal gap between the inferior aspect of the acromion and the superior aspect of the humerus (a). Postoperative anteroposterior left shoulder radiograph showing narrowing subacromial space with staple and anchors int the superior part of the humerus (b).

The surgical management of these inferior glenohumeral subluxations was proposed openly by Pinzur and Hopkins in 1986,^4^ then by Namdari and Keenan in 2010,^5^ and more recently by Thomas and Kim in 2018.^6^ This study describes an arthroscopic technique of glenohumeral suspensionplasty by biceps tenodesis, proposed for painful GHS in hemiplegic patients.

## Surgical Technique (With Video Illustration)

**Fig 2:**
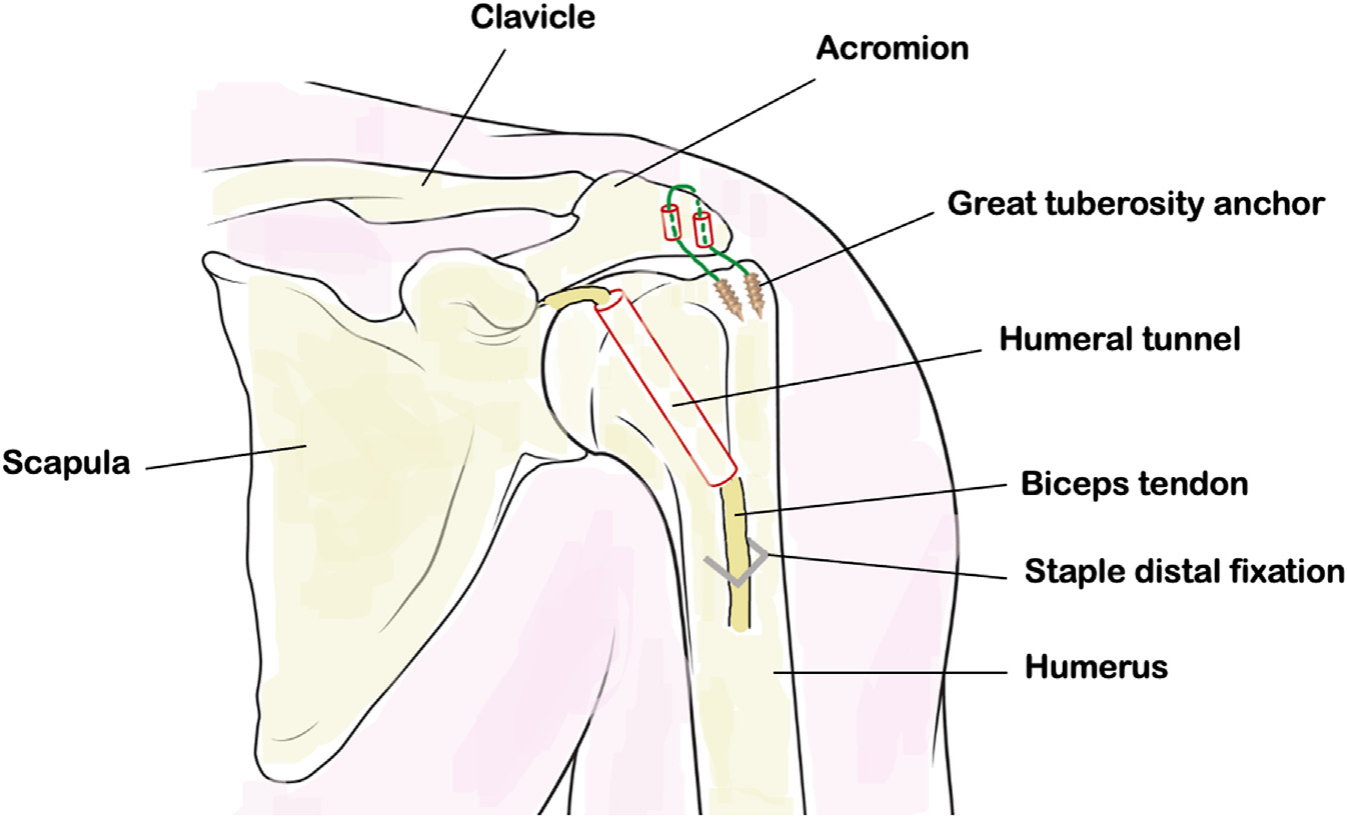
Front view of the left shoulder illustrating the arthroscopic biceps suspension procedure. A tenodesis of the long biceps on the humerus fixed by a ligament staple is performed, the long biceps tendon remaining pedicled to the glenoid, having been passed through a bone tunnel. A suspension of at least 2 anchors passed through an acromial tunnel reinforces the assembly.

A step-by-step description of the surgical technique is provided in Video 1. Figure 2 represents, in a schematic view, the surgical procedure.

### Patient Position, Arthroscopic Approaches, Exploration

**Fig 3:**
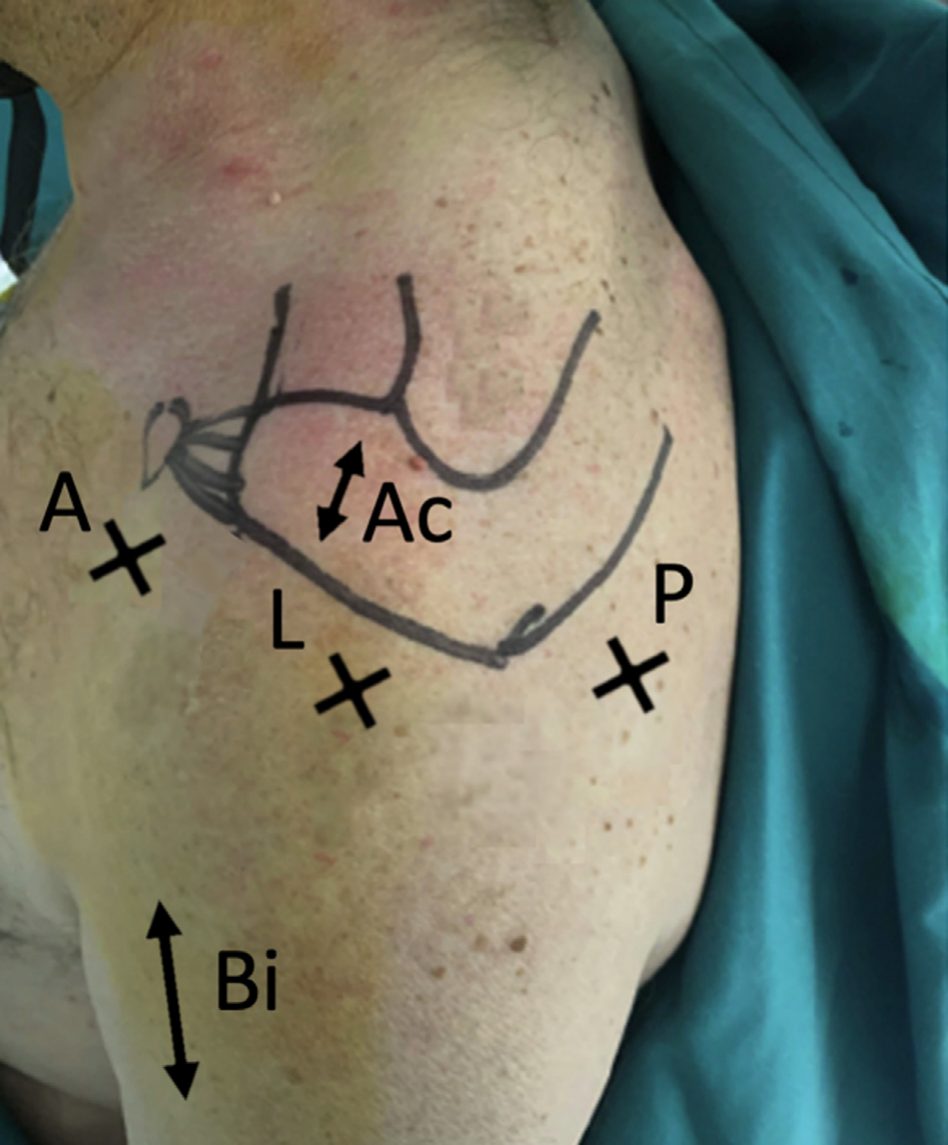
Standard arthroscopic routes in the beach-chair position for the left shoulder with posterior (P), lateral (L), and anterior (A) approaches. Bicipital groove approach (Bi) and acromial approach (Ac) are also shown.

Under general anesthesia, the patient is placed in the beach-chair position. A posterior soft-point approach is performed first to explore the joint with a 30° optic, then an anterior in-out approach in the rotator interval is performed (Fig 3). The integrity of the articular portion of the long biceps is checked carefully. A lateral arthroscopic approach will be performed secondarily to place anchors in the greater tuberosity, as well as a superior approach to the acromion.

### Harvesting the Tendon of the Long Portion of the Biceps

**Fig 4:**
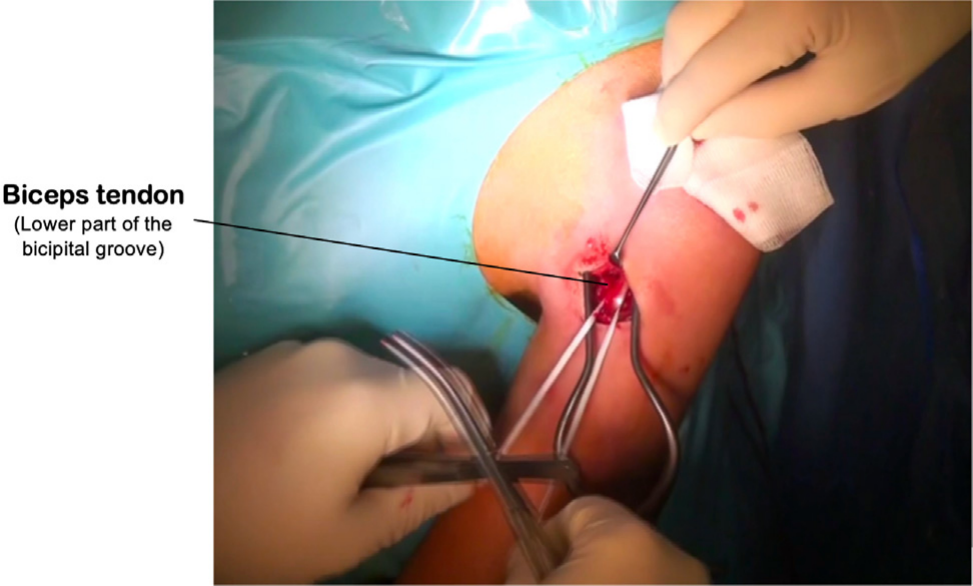
Anterior view of the left shoulder with the patient in in the beach-chair position. Open location of the long biceps tendon at the lower part of the bicipital groove, placed on a marker wire.

**Fig 5:**
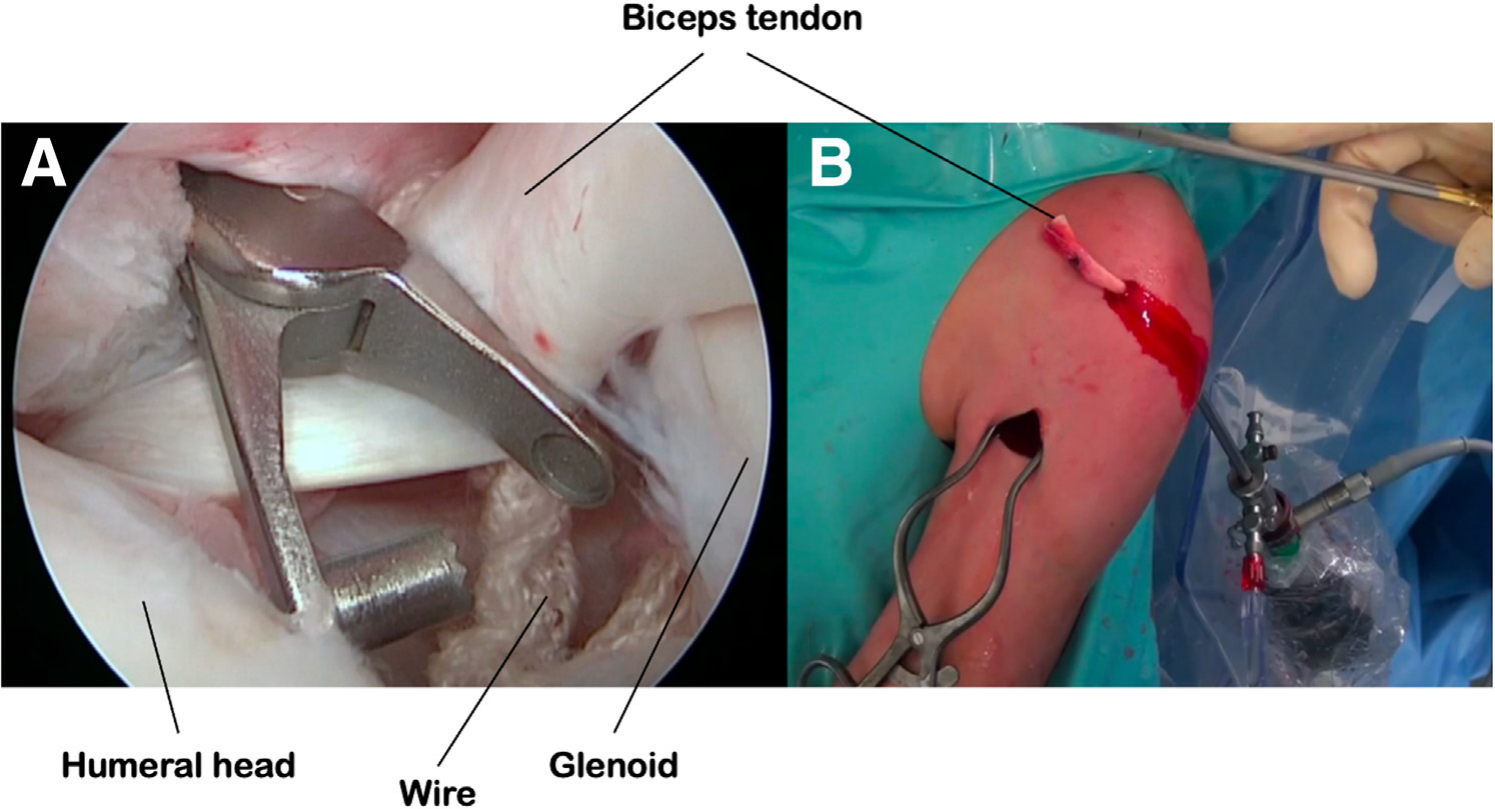
Recuperation of the long biceps tendon through the anterior arthroscopic approach using a wire passed around the tendon, scope placed in the posterior approach (a). Externalization of the long biceps tendon using the anterior arthroscopic approach (b).

A small deltopectoral incision of 30 mm is made to locate the long portion of the biceps at its exit from the bicipital groove, below the pectoralis major tendon (Fig 4). The long biceps tendon is isolated, freed, and then cut as distally as possible. In this way, the maximum length of the tendon is ensured. The tendon is then pulled proximally into the joint under arthroscopic control using a band passed around the tendon and then externalized through the anterior instrumental approach (Fig 5). Its distal end is then threaded with a Krakow suture using a number 2 PremiCron wire (B. Braun Surgical S.A., Barcelona, Spain) for easy mobilization.

### Humeral Tunneling

**Fig 6:**
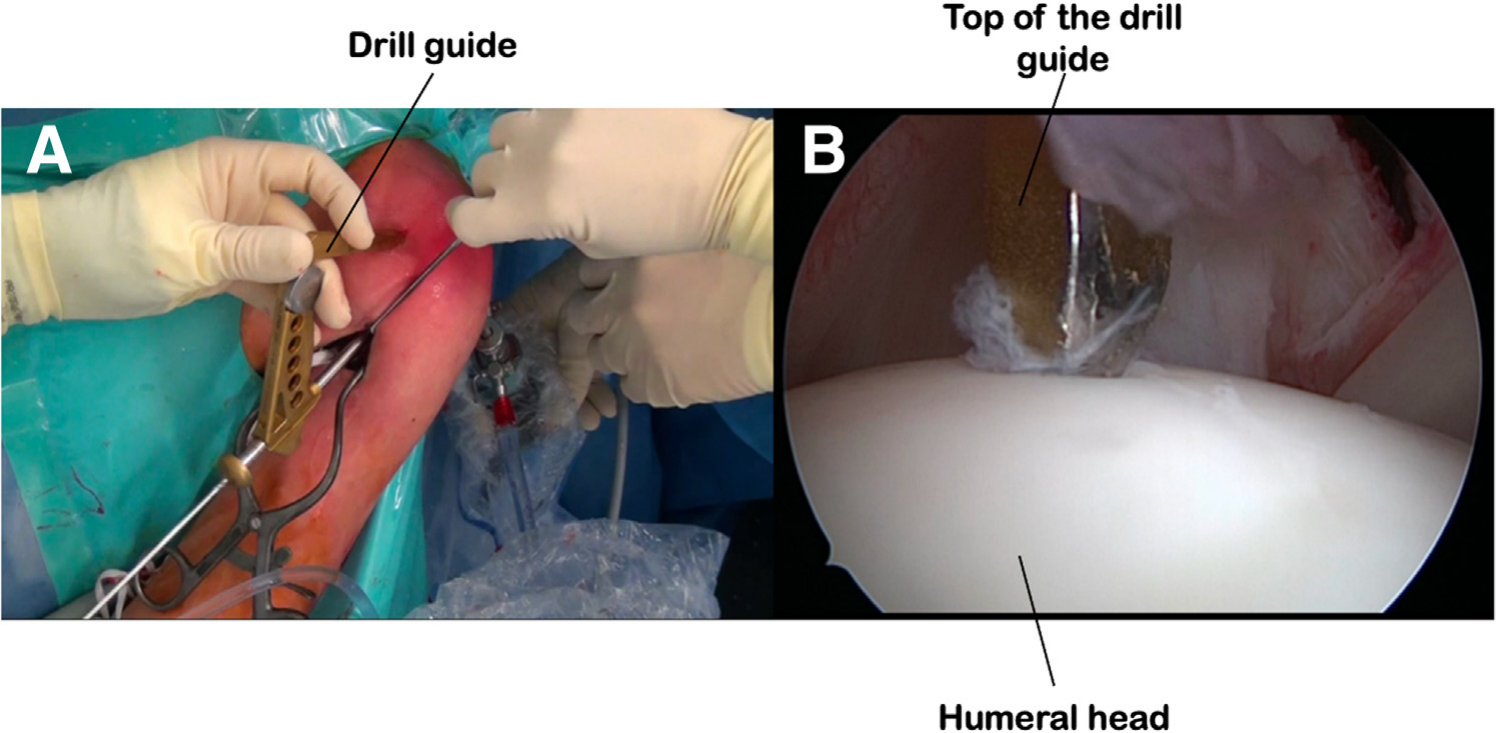
Creation of the humeral tunnel using a drill guide positioned between the anterior cortex of the humerus distally and the top of the humeral head proximally. Positioning of the guide and placement of a guidewire (a). Intra-articular position of the guide at the top of the humeral head, seen from a posterior optical approach (b).

We use a drill guide made for arthroscopic reconstruction of the anterior cruciate ligament of the knee (Smith & Nephew, Andover, MA) to create the humeral tunnel. The guide is positioned in the articulation via the anterior arthroscopic approach under the control of the optic placed via the posterior approach. The top of the humeral head is sought (Fig 6). The guide is supported at its lower part on the anterior cortex of the humeral shaft, at the level of the lower part of the bicipital groove. A guidewire is inserted and a cannulated drill bit of increasing size is used to create a tunnel to the diameter of the biceps tendon between the anterior humeral shaft and the top of the humeral head. A 10/10 steel wire is pushed through the tunnel from the shaft to the joint as a bridge wire. The biceps tendon, still attached proximally to its glenoid insertion, is pulled through the humeral tunnel by its distally attached wire and recovered at the lower part of the bicipital groove.

### Anchor Suspensionplasty

The optic is then placed in the subacromial space through a posterior approach. A lateral incision is made for instrumentation. An 8-mm arthroscopic cannula is inserted. A subacromial bursectomy is performed with a shaver. Then, a 4.75-mm SwiveLock anchor (Arthrex, Naples, FL) is inserted. The 2-wire incision is placed in the anterior part of the greater tuberosity of the humerus. A 15-mm incision at the top and middle of the acromion is made. Two vertical tunnels are drilled through the acromion from this superior approach using a 2.5-mm diameter drill bit, spaced 10 mm apart. The anterior anchor wires are pulled through the most anterior tunnel from the inside to the outside using a relay wire by a 10/10 steel wire, and then through the second tunnel from the outside to the inside by the same technique. The wire thus forms a loop on the top of the acromion and allows the humerus to be suspended from the acromion. The inferior subluxation is reduced manually, and a second SwiveLock anchor is inserted (Arthrex) is used to secure the wires under maximum tension. This is placed in the posterior part of the greater tuberosity. The purpose of this trick is to relieve tension on the biceps tenodesis during the healing phase.

### Biceps Fixation

**Fig 7:**
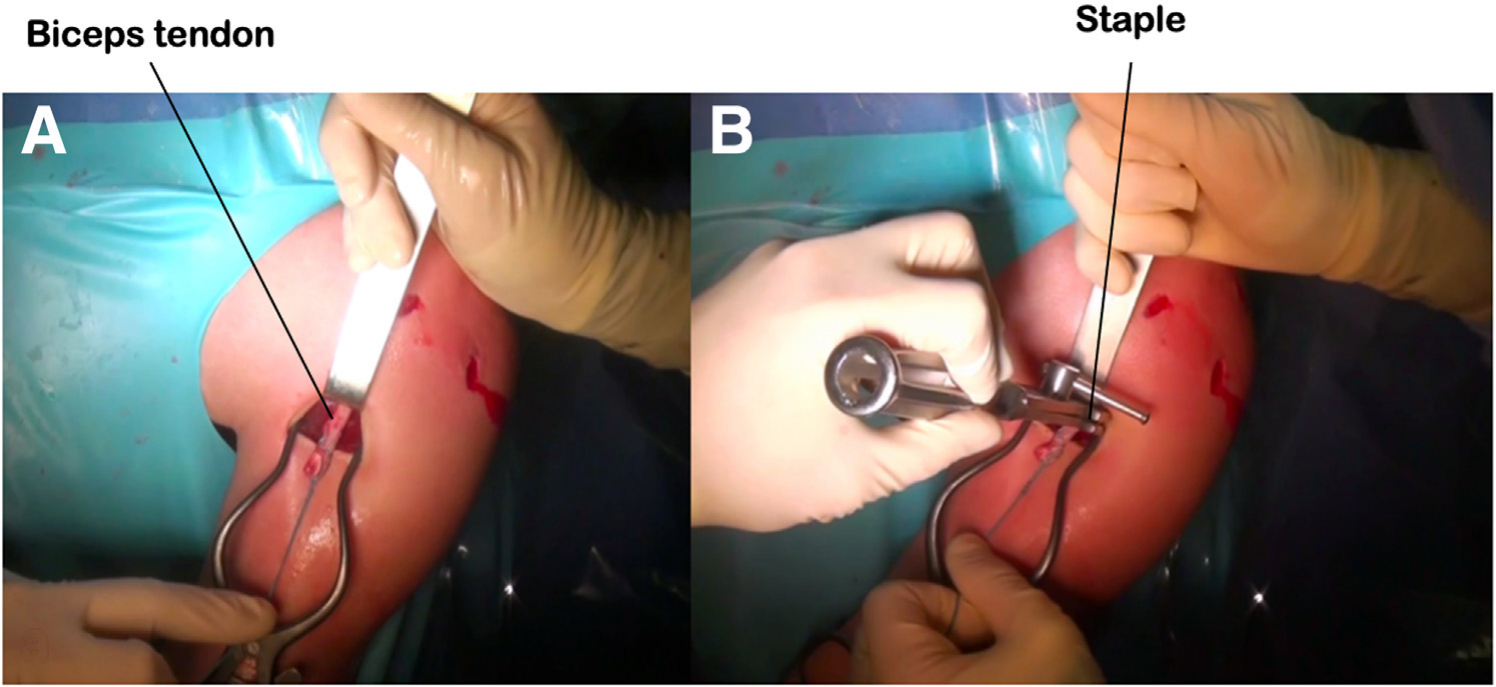
Fixation of the long biceps tendon to the humeral shaft with a ligament staple. The tendon is placed in maximum traction while maintaining manual reduction of the subluxation (a), then the staple is impacted (b).

The biceps tendon is attached to the lower part of the bicipital groove with an 11-mm wide, 25-mm long ligament staple (Lépine, Genay, France). The inferior subluxation is manually reduced and the biceps tendon is fixed in maximum tension (Fig 7). The postoperative radiograph is shown in Figure 1.

### Postoperative Care

The patient is immobilized with a sling for the first 6 weeks without any mobilization. From the end of the sixth week, the sling is gradually weaned and physiotherapy is started.

### Discussion

Although some studies report encouraging results on clinical improvement after surgical glenohumeral stabilization in hemiplegic shoulder pain,^5^^,^^6^ surgery is generally avoided because of the high anesthetic risk or morbidity associated with surgical procedures on neurologically compromised limbs. The use of a less-invasive arthroscopic technique therefore makes sense in this patient population, to decrease the morbidity of the surgical procedure, reducing scarring and septic risks.^7^

The objective of humerus suspensionplasty surgery in the patient with hemiplegia is to obtain a reduction in the inferior subluxation of the humerus in relation to the glenoid of the scapula. This requires an initial stable and durable fixation. The use of a biological stability element using the long biceps tendon as a tenodesis allows this stability over time. The use of a transosseous tunnel with intraosseous passage of the tendon allows integration of this tendon, as studied in knee ligament surgery.^8^ The combination of tenodesis with suspension by anchor and transosseous points guarantees satisfactory initial stability, reducing the risk of early relaxation of the tenodesis and recurrence of the inferior subluxation (Table 1). This dual-fixation system, tenodesis and anchor suspension, therefore, provides direct stability and ensures maintenance of stability through the biological effect of tendon integration.

**Table 1:** Pitfalls and Pearls

Pearls	Pitfalls
Use tape anchors to limit shearing of the acromion	Making the humeral tunnel too large
Use a directional support under the elbow to help reduce the subluxation	Inserting the anchors to much vertically
Start by fixing the suspensionplasty on the anchors then the biceps	

This technique is relatively simple, with low morbidity for a surgeon with experience in shoulder arthroscopy and rotator cuff surgery using common devices (Table 2). It allows for the possibility of combining repairs of intra-articular lesions (labrum, rotator cuff), common to painful GHS, in the same surgical procedure.^9^

**Table 2:** Advantages and Disadvantages

Advantages	Disadvantages
Low morbidity of the procedure	Not feasible for patients who have long head biceps tear
Possibility of diagnosing and treating associated intra-articular lesions of the shoulder	Poor anchor fixation in an osteoporotic bone
Relatively easy technique	No dedicated drilling guide for the humeral tunnel

However, the arthroscopic suspensionplasty technique has some limitations. The bone quality of patients with hemiplegia is often poor, and the anchors may be difficult to hold in osteoporotic bone. The implantation of humeral anchors must therefore be carried out in a precise manner to limit the risk of removal. For the same reason, distal biceps fixation is performed with a ligament staple, providing a satisfactory fixation in the bone. The use of interference screws could be interesting, with their reliability being shown in the subpectoral tenodesis of the biceps.^10^ Finally, this technique is not feasible in cases of rupture or significant injury of the long biceps tendon, the integrity of which must absolutely be checked at the beginning of the surgery.

## Supplementary Data

ICMJE author disclosure forms

Video 1 Video illustrating the arthroscopic shoulder biceps suspensionplasty technique.

## References

[1] Gamble G.E., Barberan E., Laasch H.U., Bowsher D., Tyrrell P.J., Jones A.K.P. (2002). Poststroke shoulder pain: A prospective study of the association and risk factors in 152 patients from a consecutive cohort of 205 patients presenting with stroke. Eur J Pain.

[2] Li Y., Yang S., Cui L. (2023). Prevalence, risk factor and outcome in middle-aged and elderly population affected by hemiplegic shoulder pain: An observational study. Front Neurol.

[3] Arya K.N., Pandian S., Vikas, Puri V. (2018). Rehabilitation methods for reducing shoulder subluxation in post-stroke hemiparesis: A systematic review. Top Stroke Rehabil.

[4] Pinzur M.S., Hopkins G.E. (1986). Biceps tenodesis for painful inferior subluxation of the shoulder in adult acquired hemiplegia. Clin Orthop Relat Res.

[5] Namdari S., Keenan M.A. (2010). Outcomes of the biceps suspension procedure for painful inferior glenohumeral subluxation in hemiplegic patients. J Bone Joint Surg Am.

[6] Thomas R.A., Kim H.M. (2018). Modified biceps suspension procedure for painful glenohumeral inferior subluxation in hemiplegic stroke patients: A preliminary study. Tech Shoulder Elbow Surg.

[7] Paxton E.S., Backus J., Keener J., Brophy R.H. (2013). Shoulder arthroscopy: Basic principles of positioning, anesthesia, and portal anatomy. J Am Acad Orthop Surg.

[8] Pinczewski L.A., Clingeleffer A.J., Otto D.D., Bonar S.F., Corry I.S. (1997). Integration of hamstring tendon graft with bone in reconstruction of the anterior cruciate ligament. Arthroscopy.

[9] Xie H.M., Zhang X.T., Xu L. (2022). Magnetic resonance imaging findings in painful hemiplegic shoulder patients with or without subluxation: A retrospective cohort study. Front Neurol.

[10] Smuin D.M., Vannatta E., Ammerman B., Stauch C.M., Lewis G.S., Dhawan A. (2021). Increased load to failure in biceps tenodesis with all-suture suture anchor compared with interference screw: A cadaveric biomechanical study. Arthroscopy.

